# Enhanced Thermal Conductivity of Polytetrafluoroethylene Dielectric Composite with Fluorinated Graphite Inducing Molecular Chain Orientation

**DOI:** 10.3390/ma18133010

**Published:** 2025-06-25

**Authors:** Qiangzhi Li, Xian Chen, Jing Zhou, Jie Shen, Wen Chen

**Affiliations:** 1 Key Laboratory of Advanced Technology for Materials Synthesis and Processing, School of Materials Science and Engineering, Wuhan University of Technology, Wuhan 430070, China; liqiangzhi@whut.edu.cn (Q.L.); 331249@whut.edu.cn (X.C.); zhoujing@whut.edu.cn (J.Z.); 2 Hubei Longzhong Laboratory, Wuhan University of Technology Xiangyang Demonstration Zone, Xiangyang 441000, China; 3 Sanya Science and Education Innovation Park, Wuhan University of Technology, Sanya 572024, China

**Keywords:** high-frequency dielectric substrates, molecular chain orientation, thermal conductivity, high-frequency dielectric properties

## Abstract

Polytetrafluoroethylene (PTFE) has been widely used as a high-frequency dielectric substrate due to its excellent dielectric properties and thermal stability. However, with its low intrinsic thermal conductivity, PTFE falls short in meeting the escalating heat dissipation demands of high-power density, high-frequency communication systems. Although the thermal conductivity of PTFE composites can be effectively improved by the high thermal conductivity fillers, it is always accompanied by a decline in dielectric properties. Molecular chain ordering is regarded as an effective strategy to improve the intrinsic thermal conductivity of polymers while maintaining dielectric properties. Unfortunately, the conventional preparation methods for ordered molecular chains, such as electrostatic spinning and uniaxial stretching, are not applicable to the preparation of PTFE substrates. In this work, fluorinated graphite (FGi) is employed to induce the in-plane orientation of PTFE molecular chains. As a result, the PTFE composite with 0.5 wt% FGi loading exhibits an in-plane thermal conductivity of 1.21 W·m^−1^·K^−1^, six times higher than the in-plane thermal conductivity of pure PTFE. In addition, this composite exhibits a superior dielectric constant of 2.06 and dielectric loss of 0.0021 at 40 GHz. This work introduces a facile method to achieve improved thermal conductivity of PTFE while maintaining its excellent dielectric properties.

## 1. Introduction

Recently, driven by the rapid development of high-power density integrated circuits for the high-frequency communication system and the Low Earth Orbit (LEO) satellite communication, there is an urgent demand for advanced high-frequency dielectric substrates with outstanding dielectric properties and high thermal conductivity (TC) [1,2,3]. Polymer composite substrates, which exhibit excellent dielectric properties and facile processability, have received much attention [4,5]. Among various polymers, PTFE has been regarded as a promising candidate due to its low dielectric constant, low dielectric loss, excellent chemical stability, and thermal stability [6,7,8]. However, its low intrinsic TC of 0.2 W·m^−1^·K^−1^ prevents its application in highly integrated electronic devices with high-power density [9].

Enhancing the thermal conductivity (TC) while maintaining the dielectric constant remains a critical challenge. Introducing high-TC fillers, such as graphene [10], hexagonal boron nitride (hBN) [11], glass fiber (GF) [12], aluminum oxide (Al_2_O_3_) [13], and aluminum nitride (AlN) [14], into the PTFE matrix is a popular approach to improve the TC of PTFE-based substrates. Unfortunately, the large amount of filler, which is required to achieve a high TC, leads to the deterioration of mechanical and dielectric properties in composites [15]. To avoid the adverse effect, optimizing the orientation of PTFE molecular chains is an alternative strategy to enhance its TC. The low intrinsic TC of polymers originates from phonon scattering on disordered molecular chains. To overcome it, some strategies have been used to optimize the arrangement of molecular chains [16,17,18,19]. For instance, stretching or electrospinning methods are utilized to achieve single-crystal polymer nanofibers with high along-axial TC [20,21]. However, these molecular chain arrangement methods are inapplicable to PTFE composite substrates due to the high melt viscosity, insolubility, and creep characteristics of PTFE. Our previous work demonstrates that the biphasic interface, determined by the shapes of the filler and PTFE crystal nucleus, can induce the crystal growth direction [22]. Thus, it is suggested that introducing fillers with lamellar structure can induce the interface-parallel orientation of the PTFE molecules at the interface, which is expected to enhance the TC of the polymer matrix.

In this work, we propose a strategy to induce the molecular chain orientation by the introduction of fluorinated graphite (FGi), whose lamellar structure and PTFE-like chemical composition render it an ideal filler for regulating PTFE molecular orientation [23,24,25]. The FGi fillers provide nucleation sites for PTFE on their surface, influence the packing of PTFE molecular chains through the steric hindrance effect of surface functional groups, and induce molecular growth along the interface. As an expected result, the PTFE composite substrates show the improved TC due to the enhanced molecular orientation of the PTFE matrix with their maintained outstanding dielectric properties.

## 2. Materials and Methods

### 2.1. Materials

Fluorinated graphite (FGi) was obtained from Hubei Zhuoxi Fluorination Technology Co., Ltd. (Xiaogan, China). Polytetrafluoroethylene (PTFE) aqueous emulsion (60 wt%, TE-3865C) was purchased from Dupont Industrial Corporation (Wilmington, DE, USA). Ethanol (analytical reagent) was provided by Sinopharm Chemical Reagent Co., Ltd. (Beijing, China).

### 2.2. Fabrication of FGi/PTFE Composites

**Figure 1:**
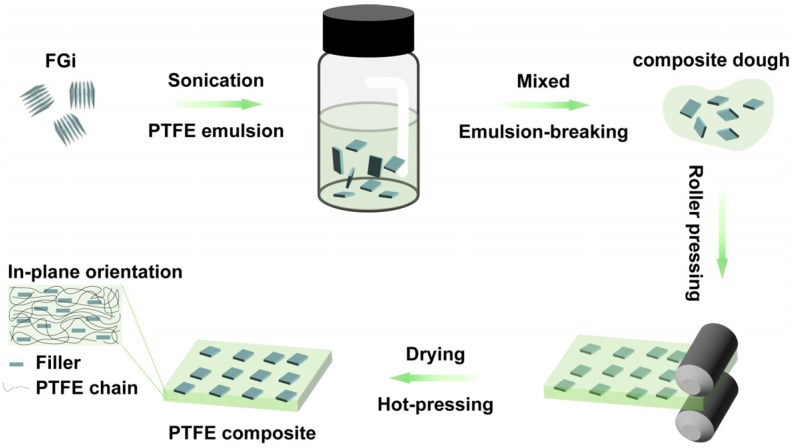
Schematic diagram of the preparation of oriented FGi/PTFE composites.

As shown in Figure 1, the preparation of FGi/PTFE composites with different FGi contents involves the following steps: First, FGi powder with the lamellar structure (Figure S1) was dispersed into PTFE aqueous emulsion via 5 min of sonication and 30 s of stirring at 1000 r/min. Subsequently, the composite doughs were prepared by dropping an appropriate amount of ethanol into the above mixtures to demulsify. The dough was repeatedly folded at room temperature and rolled into raw sheets at a linear speed of 1 m/min, followed by drying at 260 °C for 72 h to evaporate the solvent and disperser. Finally, the sheets were densified into FGi/PTFE composite substrates through hot-pressing at 385 °C under 15 MPa for 2 h, with a heating and cooling rate of 5 °C/min. The prepared FGi/PTFE composites were denominated as PTFE-*x* (*x* = 0, 0.25, 0.5, and 0.75), where “*x*” denotes the content of FGi (0, 0.25, 0.5, and 0.75 wt%) in the corresponding substrates.

### 2.3. Molecular Dynamics Simulations

In this study, the equilibrium molecular dynamics (EMD) simulations based on the Green–Kubo method are used to calculate the TC of polymeric materials to avoid the computational error due to the material size effect. EMD is carried out using LAMMPS (lammps-29AUG2024) code. The crystallinity of the model is determined by proportional mixing of the crystalline and amorphous models, while the orientation is achieved by simulating the application of a certain stress to the model. Additionally, intermolecular interactions in the PTFE system are described by the Consistent Valence Force Field (CVFF) potential function. Periodic boundary conditions are used in the EMD simulations, and the box over size is all set to 64 Å × 64 Å × 64 Å. Throughout the EMD simulation, the time step is set to 0.1 fs, and the initial velocities of the atoms are randomly sampled from a Gaussian distribution. First, energy minimization is achieved by the conjugate gradient method. The relaxation was carried out at 300 K for 2 ns under the canonical tethered system (NPT) condition, followed by another 2 ns under the canonical ensemble (NVT) condition. Finally, the TC of the calculated PTFE composites was calculated using the EMD method for the 300 K microcanonical ensemble (NVE) condition.

### 2.4. Characterization

The morphologies of FGi and FGi/PTFE composites were investigated via scanning electron microscopy (SEM, JSM-5610LV, JEOL Ltd., Tokyo, Japan). The crystalline phases of the FGi/PTFE composites were characterized by X-ray diffraction (XRD) using CuK_α_ radiation (λ = 0.15406 nm) in a Bruker D8 Advance Diffractometer (40 kV, 40 mA) (Bruker, Billerica, MA, USA). The orientation of the molecular chain was characterized by wide-angle X-ray scattering (WAXS, Xeuss 3.0 SAXS/WAXS, Xenocs, Grenoble, France). Furthermore, the melting temperature (*T_m_*) of the composites was determined by differential scanning calorimetry (DSC, DSC8500, PerkinElmer, Waltham, MA, USA) from room temperature to 380 °C at a rate of 5 °C min^−1^ in a nitrogen atmosphere. Next, in-plane and out-of-plane thermal diffusivities (TD) were obtained by the laser flash method (LFA 467 NanoFlash, Netzsch, Selb, Germany) at room temperature under atmospheric pressure. During the test, in-plane and out-of-plane fixtures were used to measure the in-plane and out-of-plane TD of the composites, respectively. The test samples were circular disks with diameters of 25.4 mm and 12.7 mm, and the laser voltage was set at 260 V with the pulse width of 100 ms. To avoid interference from the orientation of samples, the raw data in the tests were directly used without correction. The thermal conductivity (TC) was calculated from the following equation: *λ* = *α* × *ρ* × *C*p, where *λ* is the TC, *α* is the TD, *ρ* is the measured density, and *C*p is the specific heat capacity of the sample. Subsequently, the tensile properties of the composites were assessed by an electronic universal testing machine (HLD HLT, Instron 5967, Instron, Norwood, MA, USA) with a drawing speed of 10 mm min^−1^. The dielectric properties of the composites were measured by an Agilent HP8722ET microwave network analyzer (HP8722ET, Agilent, Santa Clara, CA, USA @ 1–40 GHz) with the microstrip line method according to ICP-TM-650 2.5.5.

## 3. Results and Discussion

### 3.1. Structural Characterization of the Composites

**Figure 2:**
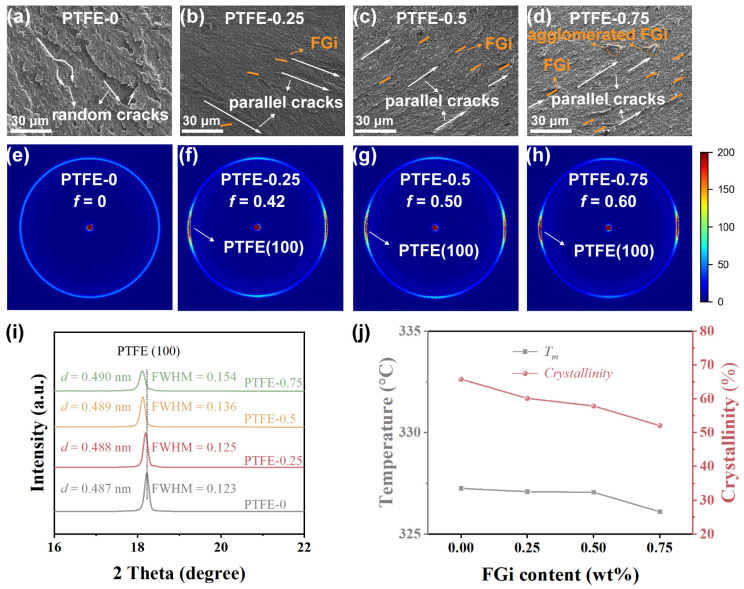
Cross-sectional SEM images of FGi/PTFE composites with FGi loading of 0 wt% (**a**), 0.25 wt% (**b**), 0.5 wt% (**c**), and 0.75 wt% (**d**), 2D WAXS patterns of FGi/PTFE composites with FGi loading of 0 wt% (**e**), 0.25 wt% (**f**), 0.5 wt% (**g**), and 0.75 wt% (**h**), XRD patterns (**i**), *T_m_* and crystallinity derived from DSC curves (**j**) of FGi/PTFE composites.

The cross-sectional SEM images of FGi/PTFE composites, which underwent brittle fracture in liquid nitrogen, are shown in Figure 2a–d. The liquid nitrogen cooling freezes the molecular chain movement during the fracture process and preserves the original orientation of the polymer matrix, which can be observed through the crack extension paths in SEM images [26,27,28]. The cracks in Figure 2a are randomly arranged, indicating the random distribution of molecular chains in pure PTFE. After the introduction of FGi, the cracks in Figure 2b–d gradually exhibit parallel alignment, attributed to the in-plane parallel arrangement of lamellar FGi fillers during rolling, which induces interfacial molecular orientation along the same direction and leads to ordered molecular chain alignment. Additionally, the roughness of the cross-section can reflect the fracture behavior of PTFE with different crystallinities, due to the fact that the fracture of PTFE occurs at the interfaces between crystalline and amorphous regions [27]. As shown in Figure 2a, the fracture surface of pure PTFE has large granularity and crystalline domain sizes. In contrast, as shown in Figure 2b–d, the size of the concave and convex regions on the cross-section of the composite decreases with FGi addition, indicating that the addition of the filler reduces the crystallinity and then the crystalline domain size of the matrix. By comparing Figure 2b–d, it can be found that as the filler content increases, the concavity and convexity undulation of the cross-section increases (the contrast in the SEM images becomes more significant). This suggests that with the increase in filler content, the entanglement between PTFE molecules intensifies, and the deformation of the matrix during fracture increases [26,28]. The orientation and steric hindrance effect of FGi enable the in-plane directional growth of PTFE molecules and reduce the crystallinity of PTFE. In addition, as shown in Figure 2b–d, due to the similar elemental composition and C-F bond, the FGi particles in the composite are tightly connected to PTFE [29]. It also can be found in Figure 2d that, at a high filler content, the filler particles in the PTFE-0.75 agglomerate, resulting in the formation of defects.

Figure 2e–h show the WAXS results used to evaluate the effect of FGi on the molecular chain orientation during the crystallization of PTFE. As shown in Figure 2e, the scattering ring of PTFE is a complete circle, indicating that the molecular chains in pure PTFE are randomly arranged in the matrix. In Figure 2f–h, the scattering rings are replaced by arcs, and the intensity increases with the increase in FGi content, which reflects the filler-induced orientation of PTFE molecules [30,31]. To quantitatively confirm the orientation of PTFE, Herman’s orientation function is used to calculate the orientation factor *f* through azimuthal scans in Figure S2.

(1) $$f=\frac{3<{cos}^{2}\theta >-1}{2}$$

(2) $$<{cos}^{2}\theta >=\frac{\int_{0}^{\pi /2}I\left(\theta \right){cos}^{2}\theta sin\theta d\theta }{\int_{0}^{\pi /2}I\left(\theta \right)sin\theta d\theta }$$

where the <*cos*^2^*θ*> is the average value of the squared azimuthal cosine for the (100) peaks of tested samples, and Ι(*θ*) indicates signal intensity at an azimuthal angle of *θ*. The results show that with the increase in FGi content, the Herman orientation factor *f* of the samples increases from 0 for PTFE-0 to 0.60 for PTFE-0.75. The increased Herman orientation factor *f* indicates that the (100) crystal plane and corresponding molecular chains of PTFE are partly ordered along the in-plane direction, which is consistent with the parallelization of cracks in the SEM images.

To investigate the effect of FGi on the crystallization behavior of PTFE, XRD and DSC tests are conducted on FGi/PTFE composites with various FGi contents. The XRD patterns in Figure 2i show that with the introduction and increasing content of the filler, the (100) interplanar spacing of PTFE increases, accompanied by an increase in the full width at half maximum (FWHM), indicating that the introduction of the filler relaxes the molecular structure of PTFE and reduces its crystallinity. It suggests that FGi restricts the crystallization of PTFE [32], which is consistent with the SEM analysis. Figure 2j demonstrates the curves of melting point (*T_m_*) and crystallinity (*X_c_*) with FGi contents, in which the *X_c_* of PTFE is calculated according to the following equation:

(3) $$X_{c}=\frac{{\Delta H}_{m}}{\Delta H_{f}\left(1-x\right)}\times 100\%$$

where *x* is the mass fraction of FGi in the composite, Δ*H_m_* is the melting enthalpy from DSC curves in Figure S3, and Δ*H_f_* is the fusion heat of completely crystalline PTFE (69 J·g^−1^) [6]. As shown in Figure 2j, the *T_m_* and crystallinity of the composites decrease with the increase in FGi content, and reach a minimum *T_m_* of 326.1 °C and crystallinity of 52% at PTFE-0.75. The decrease in *T_m_* and crystallinity can be attributed to the steric hindrance effect of the FGi, which restricts the crystallization of PTFE.

In summary, the structural characterization demonstrates that FGi can induce the orientation of PTFE molecular chains. The oriented structure enhances the propagation efficiency of phonons in the in-plane direction, contributing to the increase in TC along this direction, and thus achieving anisotropic thermal properties [33,34]. Meanwhile, due to steric hindrance effect of FGi, the crystallinity of the composites decreases. This induced effect may be driven by the interfacial interaction between FGi and PTFE during fabrication processes [35,36].

### 3.2. The Thermal Conductivity of the Composites

**Figure 3:**
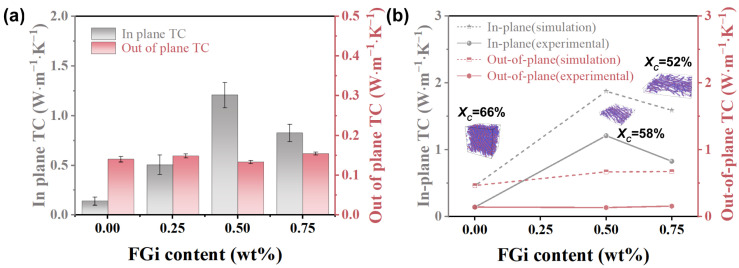
In-plane and out-of-plane TC (**a**)**,** theoretical and experimental TC (**b**) of FGi/PTFE composites.

The in-plane and out-of-plane TC of PTFE composites with different FGi contents are shown in Figure 3a. As the FGi content increases, the in-plane TC increases from 0.14 for pure PTFE to 1.21 W·m^−1^·K^−1^ for PTFE-0.5, and then decreases to 0.47 W·m^−1^·K^−1^ for PTFE-0.75. Meanwhile, the out-of-plane TC remains almost stable. In order to analyze the effect of molecular orientation and crystallinity on TC, EMD simulations based on the Green–Kubo method are used to calculate the TC of PTFE with various orientations and crystallinities. The models for various orientation degrees and crystallinity are shown in Figure S4, and the corresponding TC for each model is shown in Figure 3b. It is well known that the phonon transport efficiency in molecular chains determines the TC of polymers [20]. The oriented arrangement reduces the disordered entanglement among the molecular chains and increases the mean free path of phonons, thereby significantly enhancing the TC [17,19]. Therefore, the introduction of FGi enhances the in-plane orientation of PTFE molecules, leading to a rapid increase in the in-plane TC of PTFE-0.25 and PTFE-0.5. When the FGi content increases to 0.75 wt%, the reduced crystallinity leads to an increase in phonon scattering in the amorphous region, which results in a decrease in TC [37]. These phenomena align with the varying trends of in-plane TC observed in different models presented in Figure S5, indicating the synergistic effect of crystallinity and orientation degree on the enhancement of TC.

### 3.3. The Mechanical Properties of the Composites

**Figure 4:**
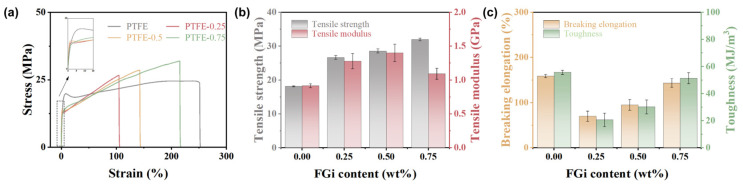
Stress–strain curves (**a**), tensile stress and modulus (**b**), breaking elongation and toughness (**c**) of FGi/PTFE composites.

Figure 4a shows the stress–strain curves of composites with various FGi contents. Compared to pure PTFE, the PTFE-0.25 and PTFE-0.5 composites exhibit reduced elasticity. This is due to the increased molecular chains entanglement caused by the decrease in crystallinity induced by the filler, which is consistent with Figure 2b,c [28]. In addition, an increase in the degree of orientation causes molecular chains to align parallel to the stress direction, enhancing interchain forces and improving the rigidity of the material. With further increase in FGi content, as shown in the inset of Figure 4a, there is no yield point in PTFE-0.75, indicating a decrease in the stiffness of the composites due to the FGi agglomeration-induced defects and low crystallinity (as shown in Figure 2d). The histograms of tensile strength and modules of FGi/PTFE composites are shown in Figure 4b. As the FGi content increases, the tensile strength gradually increases and reaches a maximum of 32MPa at PTFE-0.75, which can be attributed to the molecular entanglement effect. However, PTFE-0.75 exhibits a modulus of 1.1 GPa, lower than the 1.4 GPa of PTFE-0.5, which is due to structural defects. Notably, PTFE-0.5 exhibits enhanced tensile strength (28.6 MPa) and modulus (1.4 GPa) due to the balance between orientation and crystallinity. Figure 4c shows that both the breaking elongation and the toughness of the composites are lower than those of pure PTFE, attributable to the disruption of structural continuity by the filler [26,38]. As the increase in FGi content, the breaking elongation and the toughness of the composites increase, correlated with the enhanced entanglement of the matrix molecular chains [27,39].

### 3.4. High-Frequency Dielectric Properties of the Composites

**Figure 5:**
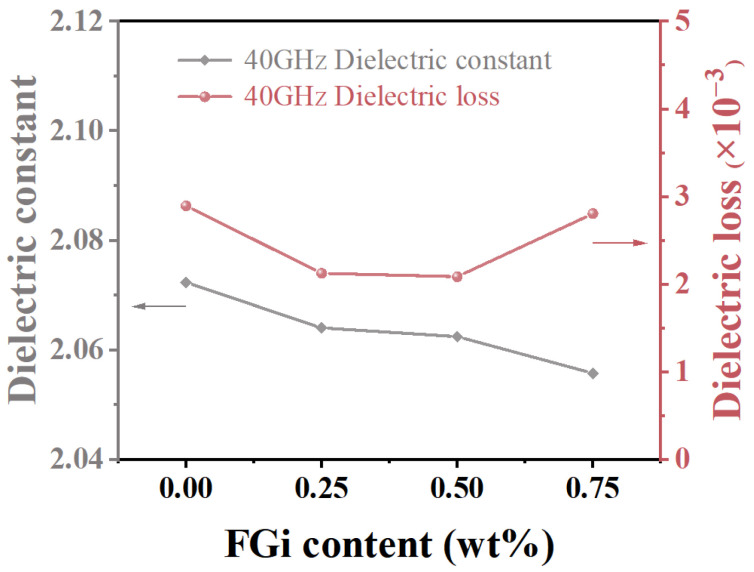
Dielectric constant and dielectric loss of FGi/PTFE composites at 40GHz.

For the substrates applied in the high frequency, the dielectric properties, including the dielectric constant and dielectric loss, are of great concern [40,41,42,43]. Figure S6 shows that the dielectric constant of the composites remains stable across frequencies from 5 to 40 GHz, indicating a highly symmetric internal structure. Meanwhile, the dielectric loss increases with frequency, which is attributed to enhanced ionic conductivity at high frequencies. As shown in Figure 5, the dielectric constant of the composites decreases with the increase in FGi content, which is due to the decrease in crystallinity of the PTFE matrix [36,44]. The dielectric loss of the composites first decreases and reaches a minimum of 0.0021 at PTFE-0.5, and then increases. The decrease in dielectric loss is initially caused by the reduced crystallinity of the composites. However, in PTFE-0.75, the aggregation of FGi sheets forms pores within the composite, which results in an increase in dielectric loss (Figure S7). The PTFE-0.5 exhibits a lower dielectric constant and dielectric loss (2.06, 0.0021@40 GHz) than pure PTFE (2.07, 0.0029@40 GHz), demonstrating its excellent dielectric properties.

In general, due to the low contents of FGi and its effects on PTFE molecular orientation and crystallinity, the PTFE-based composites retain excellent dielectric properties. These comprehensive properties significantly surpass those reported in previous studies (as shown in Table 1 and Table S1), rendering the material highly suitable for applications in high-power, high-frequency, and high-speed electronic systems.

## 4. Conclusions

In conclusion, by the introduction of FGi, highly oriented FGi/PTFE composites with enhanced TC are prepared. The in-plane orientation of the molecular chains, the primary cause of the material’s anisotropic thermal characteristics, is confirmed by WAXS and SEM observations. Highly oriented molecular chains are demonstrated by EMD calculations to be the main contribution to the increase in TC. Notably, the FGi/PTFE composite with 0.5 wt% FGi exhibits excellent TC and dielectric properties with in-plane TC of 1.21 W·m^−1^·K^−1^, dielectric constant of 2.06 (at 40 GHz), and dielectric loss of 0.0021 (at 40 GHz). Therefore, the introduction of FGi is a promising approach to enhance the TC of PTFE while maintaining the dielectric properties, which facilitates the application of PTFE composite substrates in high-frequency communication systems.

## Figures and Tables

**Table 1:** Comparison of TC and dielectric properties in PTFE-based composites.

Filler and Content	TC (W/(m·K))	Dielectric Constant	Dielectric Loss	Reference
30 vol% AlN	0.84	4.4@100 Hz	0.0035@100 Hz	[14]
32 wt% Al_2_O_3_ +21 wt% GFs	0.61	3.49@10 GHz	0.0027@10 GHz	[45]
24 wt% BNNs +1 wt% GNs	1.41	4.05@1 kHz	0.0035@1 kHz	[46]
50 vol% ZnNb_2_O_6_ +5 vol% LCP	0.88	6.46@15 GHz	0.0075@15 GHz	[47]
0.5 wt% FGi	1.21	2.06@40 GHz	0.0021@40 GHz	This work

## Data Availability

The original contributions presented in this study are included in the article. Further inquiries can be directed to the corresponding authors.

## Supplementary Materials

The following supporting information can be downloaded at: https://www.mdpi.com/article/10.3390/ma18133010/s1, Figure S1: SEM image of FGi powders; Figure S2: The azimuthal scans and Herman orientation factor *f*; Figure S3: Melt DSC curves; Figure S4: The models for various orientation degrees and crystallinity of FGi/PTFE composites; Figure S5: The in-plane TC of the model; Figure S6: Dielectric properties at various frequencies; Figure S7: Cross-sectional SEM images; Table S1: Comparison of mechanical and 10 GHz dielectric properties. References [48,49,50,51] are cited in the Supplementary Materials.
