# Supplementary material for: Enhanced Thermal Conductivity of Polytetrafluoroethylene Dielectric Composite with Fluorinated Graphite Inducing Molecular Chain Orientation
Source: Materials (Basel). 2025 Jun 25;18(13):3010. doi: 10.3390/ma18133010 (PMC12251354; doi:10.3390/ma18133010)
Supplement: Supplementary file 1 [file materials-18-03010-s001.zip › materials-3666945-supplementary.pdf]

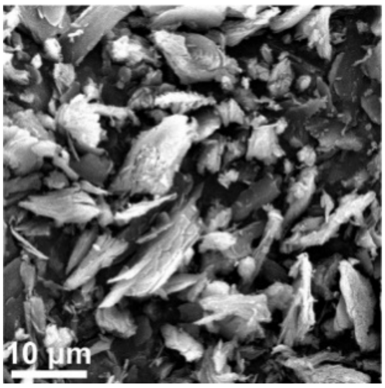

Figure S1. SEM image of FGi powders.

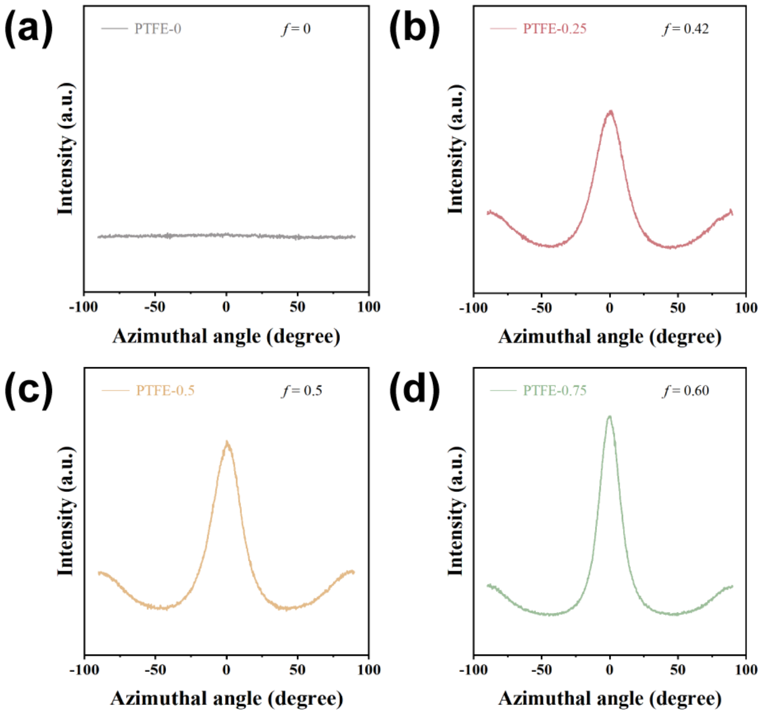

Figure S2. The azimuthal scans and Herman orientation factor f of FGi/PTFE composites with FGi loading of 0 wt% (a), 0.25 wt% (b), 0.5 wt% (c), and 0.75 wt% (d).

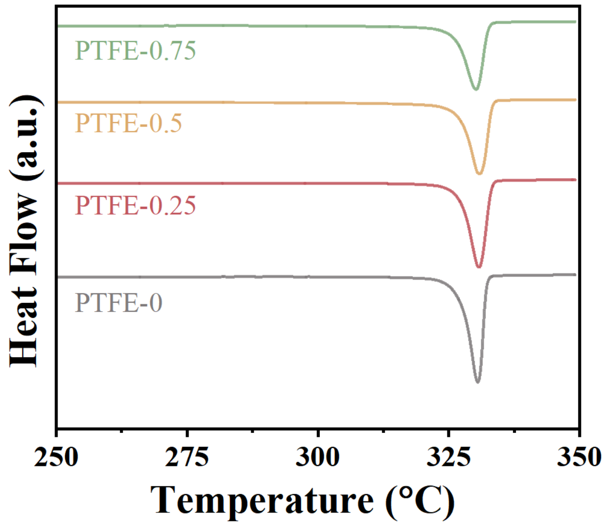

Figure S3. Melt DSC curves of FGi/PTFE composites.

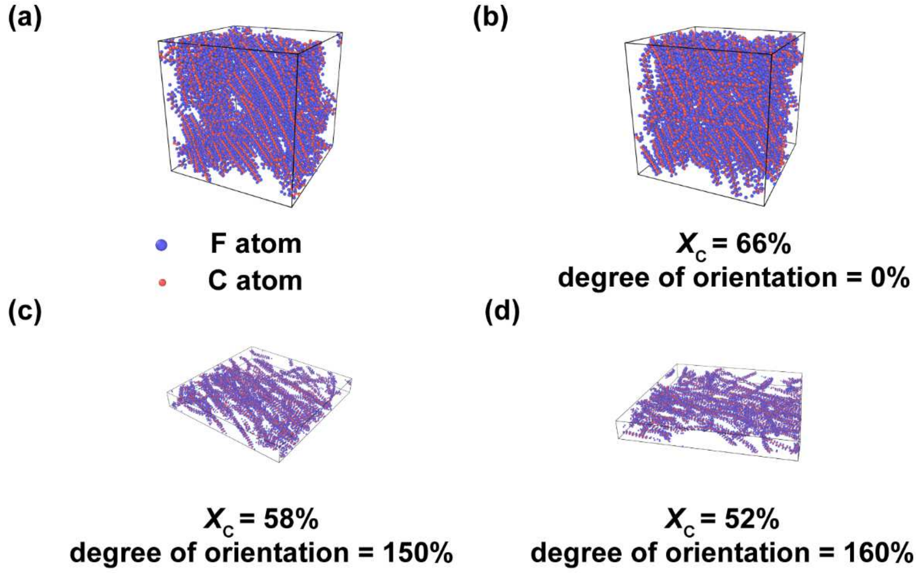

Figure S4. The original model (a), the model with 66% crystallinity and 0% degree of orientation(b), the model with 58% crystallinity and 150% degree of orientation (c) the model with 52% crystallinity and 160% degree of orientation (d).

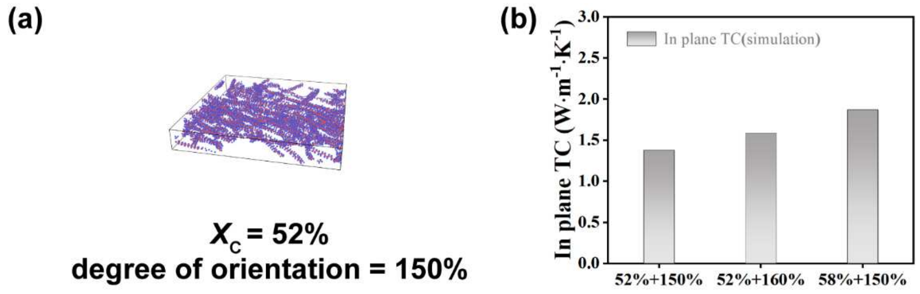

Figure S5. The model with 52% crystallinity and 150% degree of orientation (a) and the in-plane TC of the model with different crystallinity and degree of orientation (b).

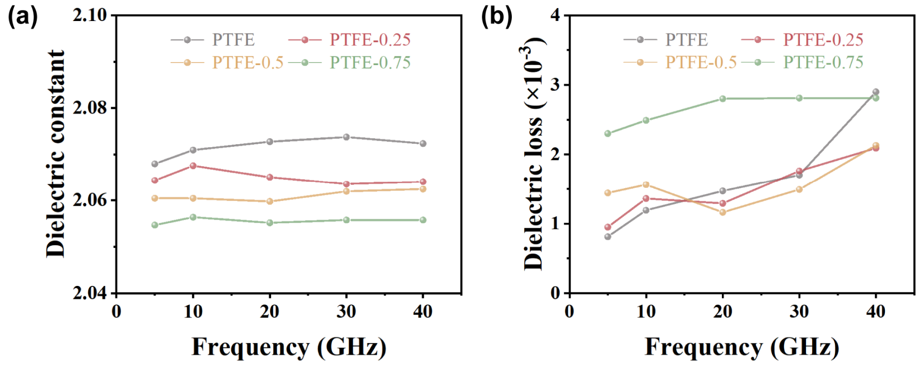

Figure S6. Dielectric constant (a) and dielectric loss (b) of FGi/PTFE composites at various frequencies.

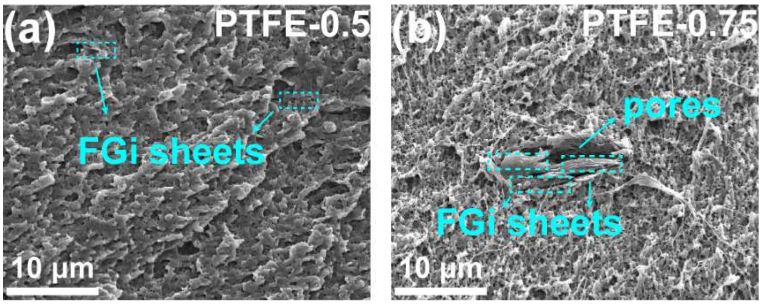

Figure S7. Cross-sectional SEM images of PTFE-0.5 (a) and PTFE-0.75 (b)

Table S1. Comparison of mechanical and 10 GHz dielectric properties in PTFE-based composites.

Filler and content	Tensile strength (MPa)	Modulus (GPa)	Dielectric constant	Dielectric loss	Reference
10 wt% SiO_2	30.7	0.5	2.15	0.0021	[1]
3 wt% PBO fibers	-	0.04	2.81	0.0138	[2]
5 wt% PI	13.7	0.3	2.09	0.0014	[3]
5 wt% Fe_2O_3	6.8	-	2.29	0.01	[4]
0.5 wt% FGi	28.6	1.4	2.06	0.0015	This work

References

1. Liu, F.; Jin, Y.; Li, J.; Jiang, W.; Zhao, W., Improved coefficient thermal expansion and mechanical properties of PTFE composites for high-frequency communication. *Compos. Sci. Technol.* **2023,** *241*, 110142.
2. He, X.; Xiao, C.; Du, H.; Wang, Y.; Ding, X.; Zheng, K.; Xue, M.; Tian, X.; Zhang, X., Significantly improved interfacial properties of silicon dioxide nanowire functionalized poly(p-phenylene-2,6-benzobisoxazole) (PBO) fibers/polytetrafluoroethylene (PTFE) wave-transparent laminated composites. *J. Mater. Sci. Technol.* **2024,** *183*, 232-240.
3. Li, R.; Liu, Z.; Chen, R.; Guo, S., In-situ fabrication of polyimide microphase and its effects on the mechanical and dielectric properties of polytetrafluoroethylene composite films. *Compos. Part A: Appl. Sci. Manuf.* **2023,** *166*. 107381.
4. Khamis, A. M.; Abbas, Z.; Azis, R. S.; Mensah, E. E.; Alhaji, I. A., Effects of recycled Fe_2O_3 nanofiller on the structural, thermal, mechanical, dielectric, and magnetic properties of PTFE matrix. *Polymers* **2021,** *13*. 2332.
